# Frequency and Predictors of Pneumonia After Isolated Coronary Artery Bypass Grafting (CABG): A Single-Center Study

**DOI:** 10.3390/diagnostics15020195

**Published:** 2025-01-16

**Authors:** Ozgur Baris, Tugba Asli Onyilmaz, Huseyin Kaya

**Affiliations:** 1 Department of Cardiovascular Surgery, School of Medicine, Kocaeli University, 41001 Kocaeli, Turkey; 2 Department of Chest Diseases, School of Medicine, Kocaeli University, 41001 Kocaeli, Turkey; asli_tugba@hotmail.com; 3 Department of Chest Diseases, Kocaeli City Hospital, 41060 Kocaeli, Turkey

**Keywords:** CABG, preoperative risk factors, postoperative pneumonia

## Abstract

**Background:** CABG is a commonly performed procedure to improve survival and quality of life in patients with coronary artery disease. Despite advances in surgical techniques and perioperative care, postoperative pneumonia remains a serious complication contributing to increased morbidity, mortality and healthcare costs. This study aims to evaluate the incidence of postoperative pneumonia (POP) and identify its risk factors in patients undergoing isolated CABG. **Methods:** This retrospective study analyzed 430 patients who underwent CABG between 2019 and 2024. Patient demographics, clinical characteristics, surgical details and laboratory data were collected. Statistical analysis included univariate and multivariate logistic regression to identify significant predictors of pneumonia. **Results:** The incidence of POP after CABG was 10% (43/430). In patients with POP, diabetes mellitus (*p* = 0.03) and chronic kidney disease (*p* = 0.048) prevalence was higher, cardiopulmonary bypass (CPB) (*p* = 0.01) and cross-clamp time (*p* = 0.003) was longer, LDH levels (*p* = 0.017) were higher, hemoglobin (*p* = 0.012) and albumin (*p* = 0.015) levels were lower, and lymphocyte % (*p* = 0.04) was lower; prevalence of COPD and length of stay (LOS) in hospital tended to be higher (both *p* < 0.06). Multivariate binary logistic regression identified COPD (OR 4.383, 95% CI: 1.106–17.363, *p* = 0.035), CPB time (OR 1.013, 95% CI: 1.001–1.025, *p* = 0.030) and LOS (OR 1.052, 95% CI: 1.004–1.103, *p* = 0.035) as independent predictors of POP. **Conclusions:** Postoperative pneumonia is a common complication after CABG and is strongly associated with preoperative COPD, CPB time and length of stay in hospital. These findings underline the importance of preoperative risk assessment and optimization. Early identification of high-risk patients may allow targeted strategies such as enhanced respiratory support and prophylactic antibiotics to reduce the incidence of pneumonia and improve clinical outcomes.

## 1. Introduction

Coronary artery bypass grafting (CABG) is the most commonly performed heart surgery worldwide, aimed at improving patients’ quality of life by alleviating symptoms of angina and heart failure while increasing survival rates. The incidence of coronary artery disease is steadily rising due to environmental and genetic factors such as lifestyle changes, diet, and air pollution, leading to a growing number of patients requiring CABG [1]. Despite improvements in preoperative, intraoperative, and postoperative care over recent decades, approximately half of cardiac surgery patients experience potentially preventable complications [2]. Infections, renal failure, atelectasis, and arrhythmias are among the complications associated with increased morbidity and mortality risks [3].

Various studies have assessed factors that predict postoperative mortality and morbidity. Development of postoperative pneumonia is one of the key contributors to prolonged intensive care unit stays, increased costs, and higher mortality rates [4]. Therefore, predicting potential pulmonary complications before surgery is crucial [5]. The effect of cardiopulmonary bypass on systemic inflammatory mediators, significant fluid shifts often leading to pulmonary edema during the perioperative period, the need for blood product transfusion, prolonged mechanical ventilation, and postoperative pain due to sternotomy or thoracotomy, all contribute to an increased risk of pneumonia. Identifying patients at higher risk for postoperative pneumonia beforehand allows for timely interventions, potentially reducing mortality and morbidity rates [6,7].

Advanced age, presence of chronic obstructive pulmonary disease (COPD), steroid use, low hemoglobin levels, and longer duration of surgery have been found to be associated with an increased risk of pneumonia after cardiac surgery [7]. However, there are still gaps in understanding how these factors interact and contribute to pneumonia risk in specific contexts, such as single-center cohorts where protocols and patient demographics may vary. Recent studies have indicated that there may be differences in the incidence of postoperative pneumonia due to heterogeneity in patient populations and care settings [1,8,9]. This study seeks to address these gaps by integrating clinical, laboratory, and surgical parameters to comprehensively evaluate pneumonia risk. By providing localized and actionable insights, the findings have the potential to inform protocol development and targeted preventative strategies, ultimately improving patient outcomes.

The aim of this study is to determine the incidence of pneumonia and the factors influencing its development after isolated coronary artery bypass grafting. By identifying patients at risk of developing pneumonia prior to surgery, it will be possible to implement preventive measures to reduce the incidence of pneumonia.

## 2. Materials and Methods

This retrospective study was conducted at a single center and included 430 patients who underwent isolated CABG between 2019 and 2024 in the Department of Cardiovascular Surgery, School of Medicine, Kocaeli University, Kocaeli, 41001, Turkey. Criteria for study participant selection are shown in Table 1.

### 2.1. Data Collection

The study analyzed various data, including demographic information (age, gender, smoking status, and comorbidities) and preoperative evaluations (e.g., left ventricular ejection fraction (EF) and European System for Cardiac Operative Risk Evaluation (EuroSCORE) scores). Details about the surgical approach—either off-pump coronary artery bypass (OPCAB) or on-pump cardiopulmonary bypass (CPB)—were recorded, along with surgical parameters such as the number of bypass grafts, CPB duration, cross-clamp time, extubation time, length of stay in the intensive care unit (ICU), and total hospital stay. Postoperative complications, including atrial fibrillation (AF), ventricular tachycardia (VT), pneumonia, and in-hospital mortality, were also evaluated. Preoperative laboratory test results (e.g., complete blood count, CRP, procalcitonin, LDH, and albumin) and microbiological findings from patients who developed pneumonia were documented.

### 2.2. Preoperative Risk Assessment

To ensure comparability of mortality rates across surgeons, institutions, and regions, mortality data were adjusted based on patients’ risk profiles. The EuroSCORE system, which has been validated as an effective predictor of early mortality following CABG surgery, was utilized for this purpose [9,10]. Logistic EuroSCORE mortality estimates calculated during preoperative evaluations were recorded for all patients [11].

### 2.3. Surgical Technique

All patients were managed according to standard protocols from surgery through discharge. CABG indications were based on 2021 ACC/AHA/SCAI Guideline for Coronary Artery Revascularization guidelines, including in patients with left main coronary artery disease (CAD) with high complexity CAD, multi-vessel CAD with diffuse CAD (e.g., SYNTAX score > 33), diabetes and multi-vessel CAD with the involvement of the proximal LAD, multi-vessel disease with reduced (<35%) left ventricular ejection fraction, and recurrent diffuse in-stent restenosis [12].

The off-pump CABG procedure, performed while maintaining normal cardiac physiology, was defined as having a ‘zero’ time for cardiopulmonary bypass (CPB) and cross-clamp and ‘no use’ of cardioplegia.

### 2.4. Definition of Pneumonia

Pneumonia was diagnosed based on the criteria outlined by the Centers for Disease Control and Prevention (CDC) and National Healthcare Safety Network (NHSN) clinical guidelines. A pneumonia diagnosis required the following [7]:

1. Evidence of new or progressive infiltrate, consolidation, or cavitation on a chest radiograph obtained at least two days postoperatively.
2. The presence of at least one symptom:
  ○ Fever (>38 °C) without an alternative explanation;
  ○ Leukopenia (<4000 WBC/mm^3^) or leukocytosis (≥12,000 WBC/mm^3^);
  ○ Altered mental status in patients aged ≥70 years without another identifiable cause.
3. Two or more of the following clinical signs:
  ○ Purulent sputum, changes in sputum quality, or increased respiratory secretions;
  ○ New or worsening respiratory symptoms (cough, dyspnea, or tachypnea);
  ○ Wheezing or bronchial breath sounds;
  ○ Worsening gas exchange (e.g., need of increased oxygen or ventilator demand).

The Local Ethics Committee of Kocaeli University approved the study in accordance with the Declaration of Helsinki. Informed consent was waived due to the retrospective design of the study, which relied on medical records.

### 2.5. Statistical Analysis

All statistical analyses were performed using IBM SPSS Statistics for Windows (version 25.0, Chicago, IL, USA). Categorical variables were expressed as numbers and percentages, while continuous variables were presented as medians or means ± standard deviations (SD). The comparison of categorical variables was performed by chi-square test. The Kolmogorov-Smirnov test was employed to assess the normality of continuous variables. Normally distributed data were analyzed using *t*-tests, whereas non-normally distributed data were assessed using the Mann-Whitney U test. Multivariate Binary logistic regression analysis (Enter method) was used to calculate odds ratios (ORs) with 95% confidence intervals (CIs). A *p*-value < 0.05 was considered statistically significant.

## 3. Results

A total of 430 patients who underwent isolated CABG were included in the study, with a mean age of 65.4 ± 8.7 years. Among these, 319 (74.2%) were male. The majority of patients (60%) were aged 65 years or older, 236 (54.9%) had a history of smoking, and 370 (86%) had comorbidities. The most prevalent comorbid conditions were hypertension (68.8%) and diabetes mellitus (44.9%). A low EuroSCORE risk was observed in 393 patients (91.4%).

Regarding surgical techniques, 318 patients (74%) underwent cardiopulmonary bypass (CPB), while 112 (26%) underwent off-pump CABG. For the CPB group, the mean CPB duration was 118.4 ± 35.8 min, and the mean cross-clamp time was 69.2 ± 21.1 min. The median extubation time was 9 h (7–13 h), while the median ICU and hospital stays were 3.5 and 8 days, respectively. Detailed demographic and surgical characteristics are summarized in Table 2.

### 3.1. Postoperative Complications

**Figure 1:**
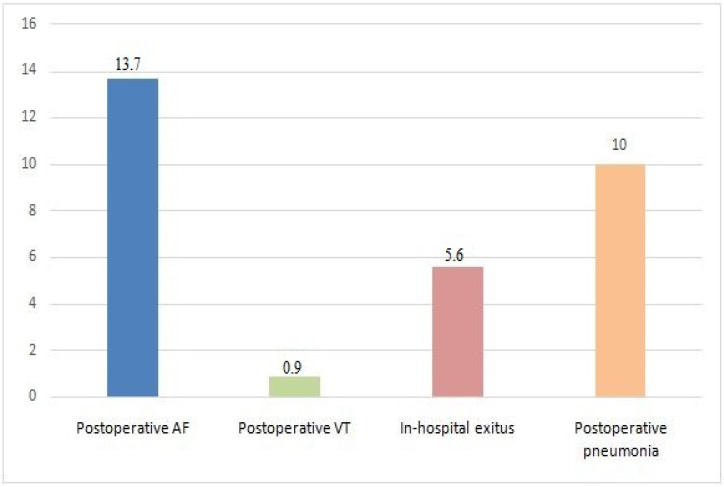
Postoperative complications of the study population (%).

**Figure 2:**
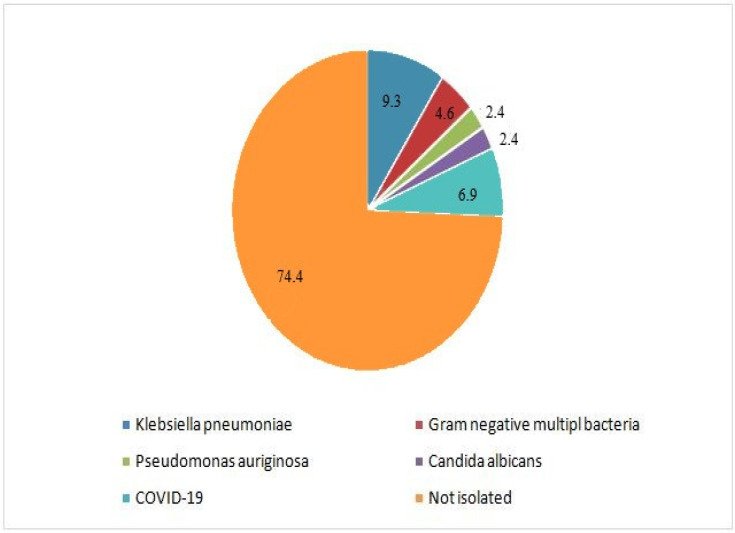
Sputum culture results (%).

Postoperative AF was the most common postoperative complication (*n*: 59, 13.7%). VT occurred in four patients (0.9%) and postoperative pneumonia was diagnosed in 43 patients (10%). In-hospital mortality incidence was 5.6% (*n* = 24), with four of these patients (16.7%) also having pneumonia (Figure 1). Among the 43 pneumonia cases, organisms were identified in 11 patients, with Klebsiella pneumoniae being the most frequently isolated pathogen. In the remaining 32 cases, no organism was identified. Sputum culture findings are presented in Figure 2.

### 3.2. Univariate Descriptive Statistics of Postoperative Pneumonia

No significant differences were observed in age or gender distribution between patients with and without pneumonia (*p* > 0.05). Comorbidities overall were not significantly associated with pneumonia; however, subgroup analysis revealed that the prevalence of diabetes mellitus (*p* = 0.03) and chronic kidney disease (*p* = 0.048) were significantly higher in patients with pneumonia than in those without pneumonia. Although the prevalence of COPD was higher among pneumonia patients compared to those without pneumonia (14% vs. 6.2%), the difference was not statistically significant (*p* = 0.058).

Surgical technique, the number of grafted vessels, and extubation time did not significantly differ between the pneumonia and non-pneumonia groups (*p* > 0.05). However, CPB time (138.84 ± 60.99 vs. 116.14 ± 31.1 min, *p* = 0.01) and cross-clamp time (79.56 ± 25.7 vs. 68.02 ± 20.3 min, *p* = 0.003) were significantly longer in the pneumonia group. Laboratory findings also indicated that patients with pneumonia had lower lymphocyte (*p* = 0.04), hemoglobin (*p* = 0.012), and albumin (*p* = 0.015) levels and higher LDH levels (*p* = 0.017). These results are detailed in Table 3.

### 3.3. Multivariate Binary Logistic Regression Analysis

A multivariate binary logistic regression analysis was used to identify the independent risk factors for postoperative pneumonia in isolated CABG patients. Variables included in the multivariate model were selected based on their significance in the univariate analysis or their clinical relevance. These variables included diabetes mellitus (DM), chronic renal disease (CRD), cardiopulmonary bypass (CPB) time or cross-clamp time, hemoglobin levels, serum albumin levels, lactate dehydrogenase (LDH) levels, lymphocyte %, chronic obstructive pulmonary disease (COPD), and length of hospital stay (LOS). To avoid multicollinearity, only one of the potentially confounding variables—CPB time or cross-clamp time—was included, prioritizing the variable deemed critical. The analysis demonstrated that COPD increased the risk of pneumonia by 4.38 times (95% CI: 1.106–17.363, *p* = 0.035). Each unit increase in CPB time was associated with a 1.013-fold increase in pneumonia risk (95% CI: 1.001–1.025, *p* = 0.030). Furthermore, a prolonged hospital stay increased the risk of pneumonia by 1.052-fold (95% CI: 1.004–1.103, *p* = 0.035). These findings are presented in Table 4.

## 4. Discussion

In this study, it was demonstrated that comorbid diseases such as diabetes mellitus and chronic kidney disease, as well as factors such as CPB time and cross-clamp time, are associated with postoperative pneumonia. A correlation was identified between the risk of pneumonia and preoperative laboratory parameters, including lower hemoglobin, albumin, and lymphocyte levels, as well as higher LDH levels. The incidence of postoperative pneumonia was found to be 4.383 times more frequent in COPD patients, 1.013 times more frequent with higher CPB time, and 1.052 times more frequent in patients with prolonged stay in hospital.

In the surgical treatment of cardiovascular diseases, coronary artery bypass graft surgery is one of the most commonly performed surgical procedures today. Serious complications such as postoperative pneumonia, which are among the comorbidities of this surgical approach, which is affected by multiple factors, can affect both short-term treatment results and long-term prognosis [13,14]. According to the literature, the incidence of postoperative pneumonia ranges from 2.62% to 21% [1,15,16]. This variation can be attributed to differences in study populations, definitions of pneumonia, diagnostic protocols, and perioperative care practices. For instance, centers employing stringent diagnostic criteria or advanced prophylactic measures may report lower rates. In our study, the incidence of postoperative pneumonia was 10%, aligning with the midpoint of the reported range. This consistency underscores the generalizability of our findings while highlighting the need for standardized reporting criteria to facilitate more accurate comparisons across studies.

Hyperglycemia and diabetes mellitus are stress-related factors that require close monitoring, especially during and after major surgeries like heart surgery. Several studies have reported that hyperglycemia is associated with increased morbidity and mortality in these cases [17,18,19]. In our study, the incidence of postoperative pneumonia was significantly higher in patients with DM.

Patients with chronic renal disease (CRD) are more prone to various complications after coronary artery bypass grafting. Particularly, advanced stages of renal dysfunction are known to be important predictors of morbidity and mortality after CABG [20,21]. In line with the literature, our study found a significantly higher incidence of postoperative pneumonia in CRD patients.

Many previous studies have reported an increased risk of postoperative pneumonia in COPD patients undergoing CABG [7,19,22,23]. In a recent meta-analysis by Gao et al., involving 37,444 patients, pulmonary complications such as postoperative respiratory failure, pneumonia, and pleural effusion were significantly more common in COPD patients compared to non-COPD patients [24]. Although our study showed a higher incidence of pneumonia in COPD patients, the difference was not statistically significant. However, in our multivariate binary logistic regression analysis, it was demonstrated that the likelihood of pneumonia was 4.38 times higher in COPD patients.

Approximately 20% of patients undergoing cardiac surgery are diagnosed with anemia prior to the operation. Anemia has been linked to an elevated risk of postoperative acute renal failure, stroke, infection, reintubation, tracheostomy, and early mortality [25]. Nevertheless, it remains uncertain whether these complications are a consequence of the anemia itself or of intraoperative or postoperative blood transfusions [26,27]. It is established that anemia affects immune cell function, with a reduction in oxygen-carrying capacity impairing macrophage and neutrophil activity. In anemic patients, reduced tissue oxygenation may have an adverse effect on postoperative recovery, potentially increasing the risk of pulmonary infections. A study by Wang et al. examining the association between anemia and postoperative complications in cardiac surgery reported that anemia increased the risk of postoperative infections, including pneumonia [28]. In our study, although serum hemoglobin levels were found to be significantly lower in the patient group with pneumonia compared to those without pneumonia, multivariate analysis did not identify it as an independent risk factor. LDH is a soluble cytoplasmic enzyme widely produced in tissues, responsible for converting pyruvate to lactate under reduced oxygen conditions. LDH is an indicator of cellular metabolism, and serum levels increase in conditions such as tissue damage, hypoxia, or inflammation. Pulmonary tissue damage or systemic inflammation could be linked to the development of pneumonia in the postoperative period. Elevated LDH levels, as markers of cellular breakdown, may reflect the presence of surgical stress or preoperative inflammatory conditions. LDH increases have been associated with acute respiratory distress syndrome (ARDS), COVID-19 pneumonia, and other pulmonary complications [29,30,31]. Similar mechanisms may be involved in postoperative pneumonia. Post-surgical tissue hypoxia and metabolic stress in cardiac surgery could trigger elevated LDH levels, which may increase the risk of complications. Our study shows that high LDH levels serve as a risk marker for postoperative pneumonia.

In our study, although multivariate analysis did not reveal a significant association, univariate descriptive analysis demonstrated that admission albumin levels were lower in the patient group with pneumonia compared to those without pneumonia. This finding suggests that low serum albumin levels may increase the risk of complications such as postoperative pneumonia. Serum albumin plays an important role in regulating plasma oncotic pressure and fluid balance, as well as modulating immune responses and defending against inflammation. Besides being a protein reservoir in the body, albumin is essential for inflammation modulation and cellular function. Since cardiopulmonary bypass is also known to induce increased inflammatory responses and cytokine levels, hypoalbuminemia may also be associated with increased risk because low albumin levels may weaken immune defenses at the cellular level, making patients more susceptible to infections [32,33]. However, low albumin levels are typically associated with clinical conditions such as malnutrition, severe inflammation, liver disease, or renal dysfunction. Each of these factors can influence the patient’s recovery capacity and increase the risk of postoperative complications. Malnutrition, especially protein and energy deficiencies, can weaken immune function, increasing the likelihood of infections like pneumonia. Additionally, during states of heightened inflammation, serum albumin levels can decrease, as albumin production is suppressed by the liver during the acute-phase response. This suggests that low albumin levels could serve as a potential biomarker for increased pneumonia risk [34].

In our study, while no significant difference in postoperative pneumonia risk was observed between surgical techniques (OPCAB/CPB), prolonged CPB time was identified as an independent risk factor. Similarly, previous studies have frequently reported an association between extended CPB duration and the development of postoperative pneumonia [6,35]. A meta-analysis conducted by He et al. concluded that longer CPB time significantly increases the risk of postoperative pneumonia [36]. These findings underscore the importance of optimizing surgical times to minimize complications such as pneumonia.

This study has several limitations. First, it is a single-center observational study. As a single-center study, it focuses on a specific patient population, making it challenging to generalize the results to other geographical or demographic groups. This may limit the applicability of the findings to a broader population. Second, the laboratory parameters were examined at the time of hospitalization and postoperative levels were not examined.

## 5. Conclusions

Our study identified several comorbidities and surgical factors associated with the development of postoperative pneumonia following CABG surgery. Preoperative parameters such as diabetes, chronic renal disease, low hemoglobin, lymphocyte, and albumin levels, and high LDH levels were associated with postoperative pneumonia. These findings emphasize the need for closer monitoring of high-risk patients and the necessity for early intervention to prevent complications such as pneumonia. Presence of COPD, prolonged stay in hospital, and CPB time were the independent risk factors for development of pneumonia. In this context, monitoring patients’ characteristics could be integrated into clinical practice for early detection and prevention of pneumonia. Identifying at-risk patients early and taking proactive measures, such as rapid extubation, optimizing postoperative invasive ventilation or administering prophylactic antibiotics, could reduce pneumonia risk in patients with CABG.

## Figures and Tables

**Table 1:** Inclusion and exclusion criteria for the study population.

Inclusion Criteria	Exclusion Criteria
Patients undergoing isolated CABG	Prior cardiac surgery Emergency CABG procedures
Age ≥ 18 years	Incomplete medical records
Complete preoperative and postoperative data	Active infection at the time of surgery Malignancy

**Table 2:** Demographic and operative characteristics of isolated CABG patients (*n*: 430).

*Demographic characteristics*		
Age, years	mean ± SD	65.4 ± 8.7
Gender, *n* (%)	Women	111 (25.8%)
	Men	319 (74.2%)
Smoking history, *n* (%)	Non-smoker	194 (45.1%)
	Current smoker	109 (25.4%)
	Former smoker	127 (29.5%)
Presence of any comorbidity, *n* (%)		370 (86%)
	Hypertension	296 (68.8%)
	Diabetes Mellitus	193 (44.9%)
	Chronic Renal Disease	36 (8.4%)
	COPD	30 (7%)
Euro SCORE, *n* (%)	Low risk	393 (91.4%)
	Moderate risk	30 (7%)
	High risk	7 (1.6%)
Preoperative EF (%)	mean ± SD	56 ± 12.4
*Surgical procedures*		
Surgical method, *n* (%)	OPCAB	112 (26%)
	CPB	318 (74%)
Number of grafts, *n* (%)	1	12 (2.8%)
	2	48 (11.2%)
	3	160 (37.2%)
	4	152 (35.3%)
	5	49 (11.4%)
	6	8 (1.9%)
	7	1 (0.2%)
CPB time, min	mean ± SD	118.4 ± 35.8
Cross clamp time, min	mean ± SD	69.2 ± 21.1
Extubation time, h	median (25th–75th percentile)	9 (7–13)
*Postoperative complications*		
	Postoperative AF	59 (13.3%)
	Postoperative VT	4 (0.9%)
	Postoperative pneumonia	43 (10%)
	In-hospital exitus	24 (5.6%)

Abbreviations: SD: Standard deviation; COPD: Chronic obstructive pulmonary disease; EF: Ejection fraction; OPCAB: Off-pump coronary artery bypass; CPB: Cardiopulmonary bypass; AF: Atrial fibrillation; VT: Ventricular tachycardia.

**Table 3:** Univariate descriptive statistics of postoperative pneumonia. Comparison of demographic characteristics, surgical procedure, postoperative complications and laboratory parameters according to the presence of postoperative pneumonia in CABG patients.

		Postoperative Pneumonia		
		(+)	(−)	*p*
*Demographic characteristics*				
Gender	Female	10 (23.3%)	101 (26.1%)	0.69
	Male	33 (76.7%)	286 (73.9%)	
Age, year	mean ± SD	65.7 ± 8.9	65.4 ± 8.7	0.82
Smoking history, *n* (%)	Non-smoker	18 (41.9%)	176 (45.5%)	0.27
	Current smoker	8 (18.6%)	101 (26.1%)	
	Former smoker	17 (39.5%)	110 (28.4%)	
Comorbidity	(+)	40 (93%)	330 (85.3%)	0.16
	(−)	3 (7%)	57 (14.7%)	
	Hypertension	34 (79.1%)	262 (67.7%)	0.13
	Diabetes mellitus	26 (60.5%)	167 (43.2%)	0.03
	Chronic renal disease	7 (16.3 %)	29 (7.5%)	0.048
	COPD	6 (14%)	24 (6.2%)	0.058
EuroSCORE, *n* (%)	Low risk	38 (88.4%)	355 (91.7%)	0.76
	Moderate risk	4 (9.3%)	26 (6.7%)	
	High risk	1(2.3%)	6 (1.6%)	
Preoperative EF (%)	mean ± SD	56.4 ± 13.04	55.99 ± 12.34	0.83
*Surgical procedure*				
Surgical method, *n* (%)	OPCAB	11 (25.6%)	101 (26.1%)	0.94
	CPB	32 (74.4%)	286 (73.9%)	
Number of grafts	1	1 (2.3%)	11 (2.8%)	0.98
	2	5 (11.6%)	43 (11.1%)	
	3	16 (37.2%)	144 (37.2%)	
	4	16 (37.2%)	136 (35.1%)	
	5	5 (11.6%)	44 (11.4%)	
	6	0	8 (2.1%)	
	7	0	1 (0.3%)	
CPB time, min	mean ± SD	138.84 ± 60.99	116.14 ± 31.1	0.01
Cross clamp time, min	mean ± SD	79.56 ± 25.7	68.02 ± 20.3	0.003
Extubation time, h	Median/(25th–75th percentiles)	9.25 (6.13–12.8)	9 (7–13)	0.87
LOS in ICU, day	Median/(25th–75th percentiles)	4 (2–5)	3.5 (3–5)	0.41
LOS in hospital, day	Median/(25th–75th percentiles)	9 (7–13)	8 (7–10)	0.053
*Post operative complications*				
	Postoperative AF	7 (16.3%)	52 (13.4%)	0.61
	Postoperative VT	0	4 (1%)	0.5
	In-hospital exitus	4 (9.3%)	20 (5.2%)	0.26
*Laboratory tests at the time of diagnosis*				
WBC, ×10^3^	mean ± SD	8.10 ± 1.98	8.31 ± 2.45	0.59
Leukocyte count, ×10^3^	Median/(25th–75th percentiles)	5 (4–6)	4.9 (3.9–6.2)	0.54
Leukocyte, %	Median/(25th–75th percentiles)	64.6 (58.3–70.8)	62 (56.9–68)	0.27
Lymphocyte count, ×10^3^	Median/(25th–75th percentiles)	1.8 (1.3–2.2)	1.9 (1.4–2.5)	0.085
Lymphocyte, %	mean ± SD	22.8 ± 7.08	25.27 ± 8.9	0.04
Hemoglobin, (g/dL)	mean ± SD	12.25 ± 2.21	13.07 ± 1.99	0.012
Platelet, ×10^3^	mean ± SD	248,095 ± 71,575	241,037 ± 67,514	0.52
MPV, (fL)	mean ± SD	9.96 ± 1.26	9.85 ± 1.16	0.57
Albumin, (g/L)	mean ± SD	37.35 ± 5.2	39.4 ± 5.2	0.015
LDH, (U/L)	Median/(25th × 75th percentiles)	231(201–316)	206 (168–265)	0.017
CRP, (mg/L)	Median/(25th × 75th percentiles)	7 (3–24)	5.6 (1.8–17.5)	0.11
Procalcitonin, (ng/mL)	Median/(25th × 75th percentiles)	0.12 (0.57–0.76)	0.12 (0.05–0.53)	0.32

Abbreviations: SD: Standard deviation; COPD: Chronic obstructive pulmonary disease; EF: Ejection fraction; OPCAB: Off-pump coronary artery bypass; CPB: Cardiopulmonary bypass; LOS: Length of stay; ICU: Intensive care unit; AF: Atrial fibrillation; VT: Ventricular tachycardia; WBC: White blood cell; MPV: Mean platelet volume; LDH: Lactate dehydrogenase; CRP: C-reactive protein.

**Table 4:** In binary logistic regression analysis, risk factors associated with postoperative pneumonia in isolated CABG patients.

Variable	OR [95% CI]	*p*
Diabetes mellitus	1.243 [0.483–3.194]	0.652
Chronic renal disease	1.904 [0.488–7.435]	0.354
COPD	4.383 [1.106–17.363]	0.035
Length of stay in hospital	1.052 [1.004–1.103]	0.035
Lymphocyte,%	1.003 [0.95–1.059]	0.913
Haemoglobin	0.801 [0.594–1.079]	0.145
LDH	1.0 [0.995–1.004]	0.898
Albumin	0.956 [0.871–1.049]	0.342
CPB time	1.013 [1.001–1.025]	0.030

Abbreviations: OR: Odds ratio; CI: Confidence interval; COPD: Chronic obstructive pulmonary disease; LDH: Lactate dehydrogenase; CPB: Cardiopulmonary bypass.

## Data Availability

Dataset available on request from the authors.
